# TRACEBACK: Testing of Historical Tubo-Ovarian Cancer Patients for Hereditary Risk Genes as a Cancer Prevention Strategy in Family Members

**DOI:** 10.1200/JCO.21.02108

**Published:** 2022-03-09

**Authors:** Rachel Delahunty, Linh Nguyen, Stuart Craig, Belinda Creighton, Dinuka Ariyaratne, Dale W. Garsed, Elizabeth Christie, Sian Fereday, Lesley Andrews, Alexandra Lewis, Sharne Limb, Ahwan Pandey, Joy Hendley, Nadia Traficante, Natalia Carvajal, Amanda B. Spurdle, Bryony Thompson, Michael T. Parsons, Victoria Beshay, Mila Volcheck, Timothy Semple, Richard Lupat, Kenneth Doig, Jiaan Yu, Xiao Qing Chen, Anna Marsh, Christopher Love, Sanela Bilic, Maria Beilin, Cassandra B. Nichols, Christina Greer, Yeh Chen Lee, Susan Gerty, Lynette Gill, Emma Newton, Julie Howard, Rachel Williams, Christie Norris, Andrew N. Stephens, Erin Tutty, Courtney Smyth, Shona O'Connell, Thomas Jobling, Colin J.R. Stewart, Adeline Tan, Stephen B. Fox, Nicholas Pachter, Jason Li, Jason Ellul, Gisela Mir Arnau, Mary-Anne Young, Louisa Gordon, Laura Forrest, Marion Harris, Karen Livingstone, Jane Hill, Georgia Chenevix-Trench, Paul A. Cohen, Penelope M. Webb, Michael Friedlander, Paul James, David Bowtell, Kathryn Alsop

**Affiliations:** ^1^ Peter MacCallum Cancer Centre, Parkville, Victoria, Australia; ^2^ Sir Peter MacCallum Department of Oncology, The University of Melbourne, Victoria, Australia; ^3^ Ovarian Cancer Australia, Melbourne, Victoria, Australia; ^4^ Prince of Wales Hospital, Randwick, New South Wales, Australia; ^5^ Royal Hospital for Women, Randwick, New South Wales Australia; ^6^ Parkville Familial Cancer Centre, Peter MacCallum Cancer Centre and Royal Melbourne Hospital, Parkville, Victoria, Australia; ^7^ QIMR Berghofer Medical Research Institute, Brisbane, Queensland, Australia; ^8^ Department of Pathology, Royal Melbourne Hospital, Parkville, Victoria, Australia; ^9^ Department of Clinical Pathology, University of Melbourne, Parkville, Victoria, Australia; ^10^ The Royal Women's Hospital, Melbourne Victoria, Australia; ^11^ The Royal Children's Hospital, Melbourne Victoria, Australia; ^12^ Department of Gynecological Oncology, St John of God Hospital, Subiaco, Western Australia, Australia; ^13^ Genetic Services of Western Australia, Perth, Western Australia, Australia; ^14^ Prince of Wales Clinical School, Faculty of Medicine, University of New South Wales, Sydney, Australia; ^15^ Prince of Wales Hereditary Cancer Centre, Prince of Wales Hospital Randwick, New South Wales, Australia; ^16^ Hudson Institute of Medical Research, Clayton, Victoria, Australia; ^17^ Department of Molecular and Translational Sciences, Monash University, Clayton, Victoria, Australia; ^18^ School of Health and Biomedical Sciences, RMIT University, Melbourne, Australia; ^19^ Monash Health Familial Cancer Centre, Clayton, Victoria, Australia; ^20^ Monash Health, Bentleigh East, Melbourne, Australia; ^21^ PathWest, King Edward Memorial Hospital, Subiaco, Western Australia, Australia; ^22^ Clinipath Pathology, Osborne Park, Western Australia, Australia; ^23^ King Edward Memorial Hospital, Perth, Western Australia, Australia; ^24^ Faculty of Health and Medical Sciences, University of Western Australia, Perth Australia; ^25^ Kinghorn Centre for Clinical Genomics, Garvan Institute of Medical Research Darlinghurst, New South Wales, Australia; ^26^ School of Nursing, Queensland University of Technology, Brisbane, Queensland, Australia; ^27^ Consumer Advocate, Melbourne, Victoria, Australia; ^28^ Division of Obstetrics and Gynaecology, Faculty of Health and Medical Sciences, University of Western Australia, Perth, Australia

## Abstract

**PURPOSE:**

Tubo-ovarian cancer (TOC) is a sentinel cancer for *BRCA1* and *BRCA2* pathogenic variants (PVs). Identification of a PV in the first member of a family at increased genetic risk (the proband) provides opportunities for cancer prevention in other at-risk family members. Although Australian testing rates are now high, PVs in patients with TOC whose diagnosis predated revised testing guidelines might have been missed. We assessed the feasibility of detecting PVs in this population to enable genetic risk reduction in relatives.

**PATIENTS AND METHODS:**

In this pilot study, deceased probands were ascertained from research cohort studies, identification by a relative, and gynecologic oncology clinics. DNA was extracted from archival tissue or stored blood for panel sequencing of 10 risk-associated genes. Testing of deceased probands ascertained through clinic records was performed with a consent waiver.

**RESULTS:**

We identified 85 PVs in 84 of 787 (11%) probands. Familial contacts of 39 of 60 (65%) deceased probands with an identified recipient (60 of 84; 71%) have received a written notification of results, with follow-up verbal contact made in 85% (33 of 39). A minority of families (n = 4) were already aware of the PV. For many (29 of 33; 88%), the genetic result provided new information and referral to a genetic service was accepted in most cases (66%; 19 of 29). Those who declined referral (4 of 29) were all male next of kin whose family member had died more than 10 years before.

**CONCLUSION:**

We overcame ethical and logistic challenges to demonstrate that retrospective genetic testing to identify PVs in previously untested deceased probands with TOC is feasible. Understanding reasons for a family member's decision to accept or decline a referral will be important for guiding future TRACEBACK projects.

## INTRODUCTION

Prevention strategies in individuals at increased genetic risk of cancer offer important opportunities to reduce cancer incidence and mortality. This is particularly relevant for tubo-ovarian cancer (referred to as ovarian cancer). Pathogenic germline variants in *BRCA1* and *BRCA2* (*BRCA1*/*2*) confer a cumulative risk for ovarian cancer by age 80 years of 44% and 17%, respectively.^1^ Other genes, including *RAD51C*, *RAD51D*, *PALB2*, *BRIP1*, and genes involved in DNA mismatch repair, are associated with a moderately increased risk of between 5% and 12%.^2-5^ In the absence of reliable early detection, prevention of ovarian cancer remains the most effective means to reduce disease impact, as the majority of patients have advanced-stage cancer at diagnosis, which is associated with high rates of relapse and mortality.

**CONTEXT**

**Key Objective**
Germline mutations in hereditary cancer risk genes, including *BRCA1* and *BRCA2*, are common in high-grade nonmucinous tubo-ovarian cancers. Retrospective identification of patients who died of their disease without being offered clinical genetic testing, which is now the standard of care, presents an opportunity to identify genetically at-risk family members before further cancer diagnoses.
**Knowledge Generated**
Our study explored three methods for the identification and genetic testing of deceased patients with ovarian cancer for pathogenic variants in hereditary risk genes. We were able to subsequently contact familial relatives of mutation carriers and offer referral for specialist genetic counseling and follow-up.
**Relevance**
Retrospective identification of pathogenic germline variants in deceased patients with tubo-ovarian cancer is feasible and importantly, results in uptake of referral to genetic services in family members. Our experiences can guide the development of similar programs internationally and address ethical and logistical challenges.

Over the past decade, two factors have positively influenced genetic testing rates of patients with newly diagnosed ovarian cancer: the demonstrated efficacy of maintenance therapy with poly (ADP-ribose) polymerase inhibitors in germline *BRCA1*/*2* carriers^6,7^ and recognition that family history–based testing criteria were frequently inadequate.^8^ Subsequently, genetic testing guidelines have been revised in many countries to include all women with ovarian cancer.^9^ There is, however, a legacy of untested, often deceased, patients whose diagnosis predated these changes. In such circumstances, the germline status of untested patients, and therefore familial risk, is likely to remain unknown until subsequent cancer diagnoses in family members, which might have been prevented had they been aware that they had inherited a pathogenic variant (PV).

Pathogenic germline variants in *BRCA1*/*2* are present in 12%-15% of patients with high-grade nonmucinous ovarian cancer (HGNMOC),^8,10^ the highest prevalence of any malignancy and therefore representing a sentinel cancer for *BRCA1*/*2* carriers. The identification of a patient with ovarian cancer within a family that is at increased genetic risk (proband) as a strategy for cancer prevention in other family members was explored at a National Cancer Institute workshop in 2016, and a conceptual framework, termed Traceback, developed.^11^ The subsequent editorial published in this journal^12^ highlighted significant ethical, legal, and social implications of retrospective identification and testing of probands and recommended pilot studies.

Here, we provide our experience in establishing and conducting, to our knowledge, the first genetic testing program for deceased patients with ovarian cancer, which we have also termed TRACEBACK. Our pilot demonstrated the feasibility and effectiveness of multiple ascertainment approaches for deceased probands, performed retrospective genetic testing, and returned significant results to families to facilitate cascade testing. We discuss the ethical and logistic challenges with this type of program.

## PATIENTS AND METHODS

This research was approved by the Human Research Ethics Committee (HREC) at the Peter MacCallum Cancer Centre (PMCC, Melbourne, Australia; HREC/17/PMCC/89) and all participating hospitals (including SJOG 1393).

### Identification of Probands

Eligible probands were patients diagnosed with high-grade nonmucinous epithelial carcinoma of the ovary, fallopian tube, or peritoneum (HGNMOC) between 2000 and 2016 and were ascertained through existing research studies (cohorts), from specialist gynecologic oncology clinic medical records (clinic), and by a next of kin (NOK) of a deceased HGNMOC proband (NOK referral). Further details of case ascertainment are provided in the Data Supplement (online only). Probands determined to have previously undergone *BRCA1*/*2* germline testing in a clinical or research setting were excluded.

### Vital Status

Although there was provision within the TRACEBACK study to facilitate testing of living probands (Data Supplement), here, we evaluate the implications of genetic testing in the deceased ovarian cancer population.

### Genetic Testing

**FIG 1.**
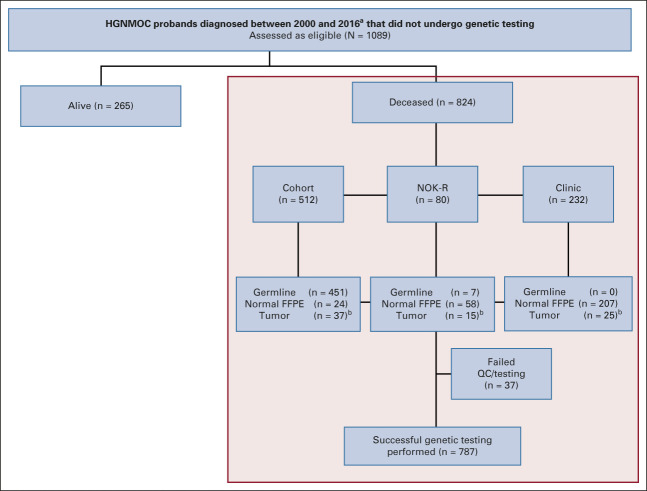
TRACEBACK probands and samples. Schematic representation of the three ascertainment methods used to identify and test high-grade nonmucinous ovarian cancer probands, diagnosed in Australia between the years 2000 and 2016. Genetic testing of deceased probands (n = 824) is reported here, with information available for the living probands ascertained to the study (n = 265) in the Data Supplement. ^a^Some exceptions were made for deceased probands diagnosed outside 2000-2016 for NOK referral (Data Supplement). ^b^Predominately normal tissue was obtained from surgical tumor samples and included both FFPE and fresh-frozen biospecimens (Data Supplement). FFPE, formalin-fixed paraffin-embedded; HGNMOC, high-grade nonmucinous ovarian cancer; NOK-R, Next of Kin–referred; QC, quality control.

Germline DNA derived from blood was available for most cohort-ascertained probands. If unavailable, we obtained DNA extracted from either frozen or formalin-fixed paraffin-embedded (FFPE) tissue (Fig 1 and Table 1). A gynecologic pathologist reviewed all tissue, and DNA was extracted from areas enriched by needle dissection for normal content where possible. In some instances, only tumor-rich tissue was available (Fig 1 and Table 1).

**TABLE 1.**
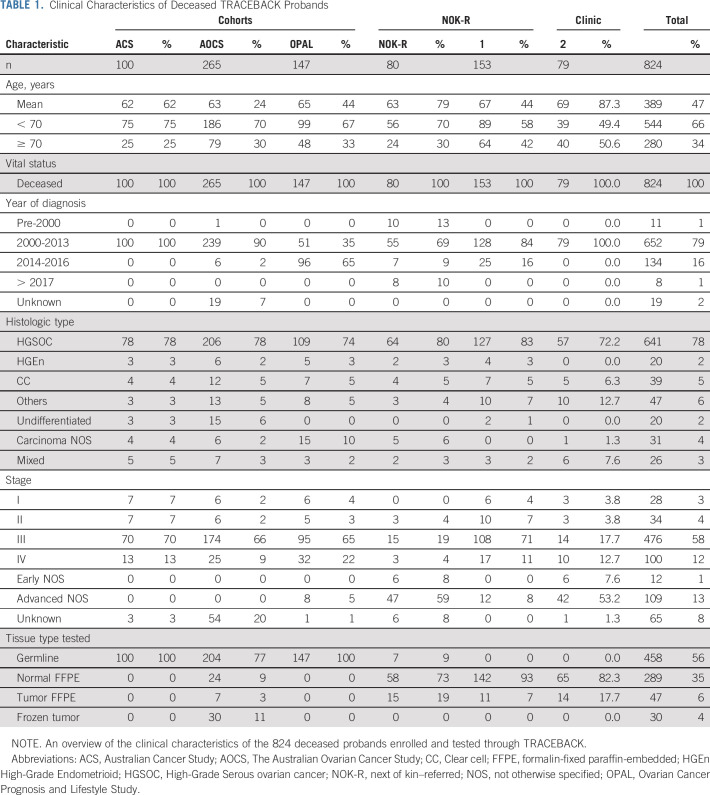
Clinical Characteristics of Deceased TRACEBACK Probands

Genetic testing was performed using a customized Agilent SureSelect^XT^ Low Input capture panel (Design ID 314187) of 79 genes (Data Supplement). Libraries were prepared using the SureSelect^XT^ Low Input Target Enrichment System (Agilent, Santa Clara, CA) and sequenced on an Illumina NextSeq 500 (Data Supplement).

### Variant Detection and Curation

Sequence alignment and variant detection are described in the Data Supplement. Ten genes (*BRCA1*, *BRCA2*, *RAD51C*, *RAD51D*, *BRIP1*, *PALB2*, *MLH1*, *MSH2*, *MSH6*, and *PMS2*), regarded as clinically actionable,^9^ were reviewed in PathOS^13^ and single-nucleotide variants and indels filtered to remove synonymous and common variants (global minor allele frequency > 1%). Potentially pathogenic nonsynonymous variants were reviewed manually via Integrative Genomics Viewer.^14,15^ Variant pathogenicity was assigned using databases such as ClinVar,^16^ InSiGHT,^17^ and literature from expert panels^18^ and classified according to the International Agency for Cancer Research (IARC) and American College of Medical Genetics and Genomics (ACMG).^19,20^ All variants of unknown significance (C3), likely pathogenic/pathogenic (C4/C5) variants or any with ambiguous classification, were committee reviewed. Variants determined to be C4/C5 (here after referred to as PVs) were subsequently validated in a National Association of Testing Authorities, Australia (NATA; ISO15189)-accredited clinical laboratory, using independent DNA extractions where available (Data Supplement).

### Return of Findings

A familial recipient for the research results was identified from the proband's medical records or the research cohort consent form (Data Supplement). Results were disseminated using a two-step process. This involved delivery of a written notification (Data Supplement) alerting the recipient of genetic information availability without specifying findings, followed by a telephone call from a genetic clinician (counselor/geneticist or a knowledgeable specialist known to the family; GC). During follow-up contact, referral to a local Familial Cancer Clinic (FCC) for predictive testing was offered.

### Statistical Analyses

Data were analyzed using GraphPad Prism 8.4 for Windows (GraphPad Software, San Diego, CA) or in the R software. A *P* value of .05 was considered statistically significant (further details are given in the Data Supplement).

## RESULTS

### A Large Population of Undetected PV Carriers

Approximately 17,000 women were diagnosed with invasive HGNMOC in Australia between 2000 and 2016.^21,22^ On the basis of the reported testing rates from this period in two Australian studies^8,23^ and data captured by participating research studies, we estimated that 12,000 patients missed genetic testing. Incorporating survival data, we anticipated that approximately 60% would have died of their disease by 2018.^24^ Previous literature indicates that 15% of Australian patients with HGNMOC carry a pathogenic germline *BRCA1*/*2* variant.^8^ We therefore estimated that in 2018, approximately 1,100 *BRCA1/2* pathogenic germline variant carriers exist among untested, deceased patients with ovarian cancer in Australia.

### Three Approaches to Proband Ascertainment

We used a multimodal approach for proband ascertainment (Data Supplement). The most straightforward involved accessing specimens from national ovarian cancer research studies. TRACEBACK was also open to referral of a deceased HGNMOC proband by a family member (NOK referral), who would otherwise be unable to access Australian government–subsidized genetic testing as it is currently restricted to living probands. We encouraged FCCs and clinicians to refer relatives who may be interested in participating. These individuals provided consent for genetic testing of their family member's tissue.

We recognized that the most efficient way to identify large numbers of eligible probands would be through auditing the medical records of primary treatment centers as most ovarian cancer surgery in Australia is centralized to metropolitan tertiary hospitals. Local clinical investigators reviewed medical records for eligible probands, including determination of previous genetic testing and vital status. In consultation with Institutional Review Boards (IRBs), we carefully considered whether to seek consent from relatives before testing. The Australian National Health and Medical Research Council (NHMRC) National Statement on Ethical Conduct in Human Research^25^ allows a waiver of the requirement for consent in certain circumstances. The rationale for proceeding with testing under a consent waiver and the processes put in place to minimize harm are detailed in the Data Supplement and Discussion.

### PVs

We report results for the first 824 deceased probands, including 512 cohort-ascertained, 80 self-referrals, and 232 ascertained from participating clinics (Fig 1). Blood-derived DNA was available for 88% (451 of 512) of the cohort probands, with normal FFPE tissue used for 85% (265 of 312) of clinic/NOK-referral probands and tumor DNA for the remainder (Fig 1). Clinical features are summarized in Table 1.

**FIG 2.**
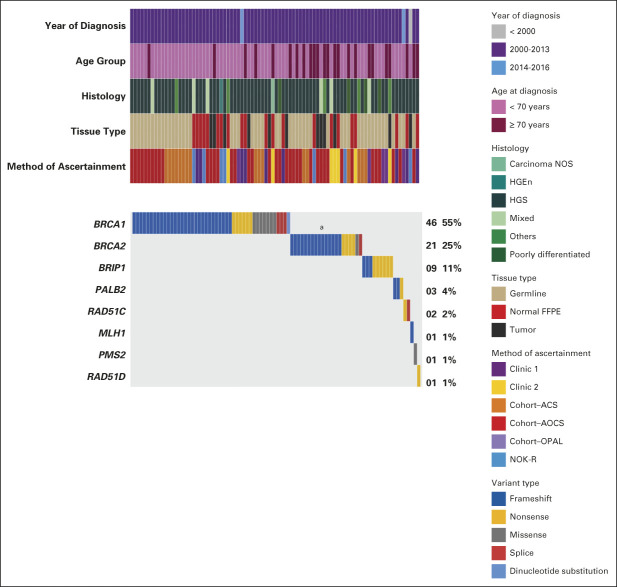
Detected C4/C5 variants (PVs). An overview of the clinical and genomic features of 84 of 787 (11%) deceased probands with PVs, including one proband with two PVs, both in *BRCA2* (one frameshift and one nonsense [given in ^a^]). ACS, Australian Cancer Study; AOCS, Australian Ovarian Cancer Study; Carcinoma NOS, Carcinoma not otherwise specified; FFPE, Formalin-fixed paraffin-embedded; HGEn, High-Grade Endometrioid; HGNMOC, high-grade nonmucinous ovarian cancer; HGS, High-Grade Serous; NOK-R, Next of Kin–referred; Opal, Ovarian Cancer Prognosis and Lifestyle Study; PV, pathogenic variant.

**FIG 3.**
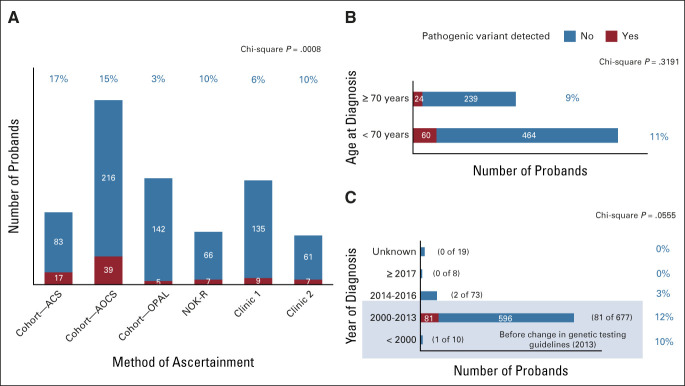
Frequency of PVs in the 787 deceased probands stratified by (A) method of ascertainment, (B) age at diagnosis, and (C) years of diagnosis. Group differences for categorical variables were examined using the chi-square test (GraphPad Prism 8.4 for Windows). ACS, Australian Cancer Study; AOCS, Australian Ovarian Cancer Study; NOK-R, Next of Kin–referred; OPAL, Ovarian Cancer Prognosis and Lifestyle Study; PV, pathogenic variant.

After sequencing, 96% (787 of 824) of samples met predefined quality control measures (Data Supplement). Eighty-five PVs were detected in 84 probands (84 of 787; 11%; Fig 2). *BRCA1*/*2* had the highest frequency of PVs (46 of 85, 54% and 22 of 85, 26%, respectively; Fig 2) with variability depending on the proband source, patient age, and year of diagnosis (Fig 3). Consistent with previous findings,^8^ *BRCA1*/*2* PVs were predominantly frameshift variants in the largest coding exons (Fig 2 and Data Supplement). Twenty percent (17 of 85) of PVs occurred in genes other than *BRCA1*/*2*, including *BRIP1* (9 of 85; 11%) and *PALB2* (3 of 85; 4%). Two PVs (2%) were detected in Lynch syndrome–associated genes (Fig 2 and Data Supplement).

All PVs identified were independently validated (Data Supplement). Thirteen PVs were initially detected in tumor-derived DNA, including one case with two *BRCA2* PVs (Data Supplement). For the purposes of validation, we were able to enrich the independent DNA for normal tissue content in 5 of 12 cases (Data Supplement). PVs in the remaining seven cases were validated with tumor DNA, and the possibility of somatic occurrence is recognized. Sixteen percent (126 of 787) of probands had a variant of unknown significance detected (Data Supplement), which were not validated or returned because of uncertainty of their clinical significance.

### Recipients for Notification of Genetic Test Findings

**FIG 4.**
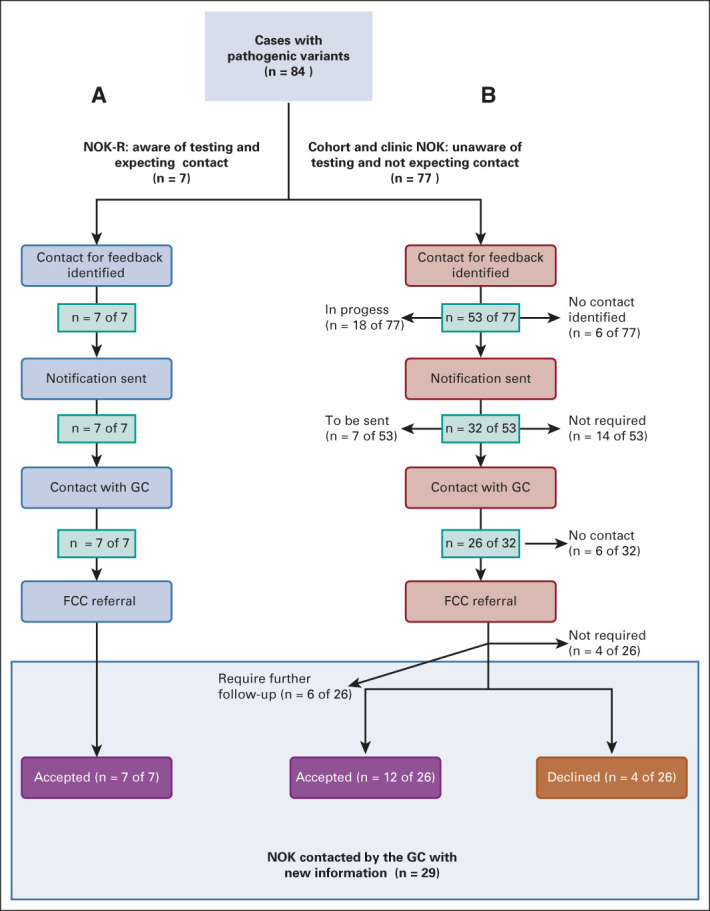
Identification of recipients for research results. A stepwise representation of the feedback of PVs to date: (A) outcome of the feedback process to the NOK of deceased probands who self-referred into the study and (B) outcome for feedback to 77 NOKs of deceased probands ascertained via clinic-based recruitment and existing research studies. Created using BioRender. GC, Genetic clinician; NOK-R, next of kin–referred; PV, pathogenic variant.

To date, an appropriate familial recipient (next of kin; NOK) with verified contact details for the notification of a PV was identified for 71% (60 of 84) of the deceased probands (Fig 4 and Data Supplement). Despite a thorough search, no appropriate contact could be found in six instances (7%) and identification of an appropriate recipient of findings is ongoing for the remainder (18 of 84; 21%).

The study commenced in 2018 and was cross-checking with updated clinical information before notifying results, and 14 of the 60 probands with an identified contact were found to have subsequently received clinical genetic testing since initially identified as eligible (Data Supplement). In each case, the findings of this study were consistent with the result ascertained through clinical testing and notification of research results was not required in these instances. To date, of the remaining 46 of 60 probands with confirmed recipient details for results, 85% (39 of 46) have been sent a written notification (Fig 4 and Data Supplement). This includes seven NOKs that self-referred to TRACEBACK and familial contacts for 32 cohort or clinic-ascertained probands.

### GC Manages Processes After Initial Notification

Approximately two weeks after the notification letter was sent, a GC contacted the identified recipient. To date, verbal contact has been made with 86% (33 of 39) of individuals to whom a notification has been sent (Fig 4 and Data Supplement). Attempted verbal contact has been made in the remaining six cases.

The feedback of PVs to NOK recruited to TRACEBACK via NOK referral (7 of 33; 21%) was relatively simple and successful, and all accepted an FCC referral (Fig 4 and Data Supplement). By contrast, returning results to the NOK of the deceased cohort and clinic-ascertained probands (26 of 33; 79%) was more challenging. Follow-up contact by the GCs to the NOK was unexpected and, in most cases, occurred years after the proband's initial diagnosis, or death. Contact between the GC and NOK was challenging in some instances, for example, to convey to the NOK in a short period of time the significance of an unexpected phone contact. However, in most instances (24 of 26; 92%), a meaningful discussion resulted. Overall, 55% (12 of 22) of NOK presented with new and unsolicited genetic information have already accepted a FCC referral (Fig 4). Of the remaining NOKs, four were already aware of the familial variant, two declined further clinical support, and six required further input; this included three instances where it was requested that contact is made with an alternative family member and three NOKs requested more time to consider the implications of the findings for their family. Finally, in the remaining two instances (2 of 26; 8%), although an initial verbal contact was made, further communication attempts with the NOK were unsuccessful and interpreted as a decline of the offered information. All declining NOKs were male, and their proband family member had died more than 10 years before.

## DISCUSSION

Detection of pathogenic germline variants in *BRCA1*/*2* or other risk genes is relevant to the treatment of patients with ovarian cancer and provides an opportunity for cancer prevention in family members. Although identifying genetic predisposition in a family can cause distress, for most, it is seen as an opportunity to reduce cancer risk.^26^ Many women diagnosed with ovarian cancer died before genetic testing criteria became less restrictive, representing missed opportunities for cancer prevention in family members. The value of assessing such patients relies on feasibility, accurate testing, and uptake of risk-reducing strategies in recipients of significant results.

Although conceptually simple, TRACEBACK presented significant ethical and logistic challenges. We explored three different proband ascertainment pathways, recognizing that ascertainment through specialist clinics provided an opportunity to capture a large proband population, independent of socioeconomic and geographical factors, or previous opportunities to participate in research. It was, however, also the most ethically challenging.

TRACEBACK is, to our knowledge, not only the first program of its kind internationally but also the first study to perform research genetic testing for clinical applications under a consent waiver. Although decisions regarding genetic testing should be autonomous, our intention to focus on deceased probands precluded personal consent, aside from historical unspecified research consent for a proportion of probands. We recognized that contacting family members before the use of their deceased relative's sample, of which only a minority would be determined to harbor a pathogenic germline variant, would greatly slow progress of the study, reduce the proband population, and likely render the study unfeasible. Instead, we proposed limiting contact only when a PV was identified. This targeted approach reduced the challenge of identifying an appropriate NOK by an order of magnitude and prevented causing unnecessary distress in a majority of families that are not at increased genetic risk of cancer. Working within the Australian NHMRC research guidelines, and in consultation with our IRB, we balanced the risk of causing distress through the receipt of unrequested information against the potential for cancer prevention. In considering these factors, we drew on prior experience of acceptability of genetic testing information within families.^26,27^ In addition, a previous Australian study using immunohistochemistry as a surrogate marker of Lynch syndrome among unconsented patients with colorectal cancer was informative, as a majority of recipients found the unsolicited information valuable.^28^ We further mitigated the potential of harm caused by the delivery of unwanted information through graded feedback of findings involving a GC,^29^ designed to highlight the existence of important health information but at the same time providing the recipient some control of how much and what information is disclosed to them.

A positive reception of the research results by family members is critical for the success of TRACEBACK programs. NOKs of deceased probands who self-referred were, as expected, highly engaged with the project, irrespective of their sex, and all accepted referral to an FCC after notification of clinically relevant results by the TRACEBACK GC. To date, where the TRACEBACK research result was new, 55% (12 of 22) accepted a referral. This favorable rate occurred at a time of added disruption to health services and increased complexity of daily life for many individuals because of the COVID-19 pandemic.

When contacted unexpectedly with new, and unsought, genetic information, female NOKs were more likely to accept a FCC referral 63% (5 of 8) compared with male NOK 53% (7 of 14). This is consistent with other studies that demonstrate that men are generally less engaged with genetic research and less likely to have testing.^30,31^

Further work is planned to understand the factors influencing decision making around the uptake of research-generated genetic information and exploring steps that family members take to reduce their risk. The success of such genetic testing programs, particularly those focused on cancer syndromes that are primarily recognized as affecting females, may be improved by focusing on female family members as the preferred recipient of clinical follow-up. The delivery of unexpected genetic information years after the death of a family member can be confronting, and considerable care is required. The outcome of a potentially difficult interaction may be more successful when conducted by a clinician who is known and trusted by the family.

The molecular findings are similar to previous investigations in HGNMOC,^10^ supporting our technical approach. The frequency of *BRCA1*/*2* PVs in the TRACEBACK cohort is at the lower end of that in other population-based data sets,^8,10^ likely because of an ascertainment bias that excluded probands who had previous genetic testing. For technical reasons, our analysis to date has not included exploration of copy number variants that are expected to contribute a small number of additional PVs.^8,32^ The majority of cohort-ascertained probands had stored blood samples that were able to be accessed for testing purposes and, as expected, performed well in the assay. In the absence of blood-derived DNA, 93% of DNA samples derived from the use of non-neoplastic FFPE tissue passed our quality control measures (Data Supplement). Despite pathologic review and efforts to enrich for non-neoplastic tissue, PV detected and validated in DNA extracted from tumor tissue may indeed have somatic changes detected in contaminating tumor cells. Such results were nevertheless deemed notifiable by the variant review committee and were useful for relatives, who could then undergo funded personal predictive testing.

How far into the past should probands be ascertained for testing? We were successful in achieving a result in the majority of sequenced cases (96%), irrespective of the age of the tissue. However, in general, the longer it has been since a patient has been diagnosed, the more difficult it is to find a NOK and the greater the chance that new cancers have already arisen in the family and genetic risk has become apparent. In this pilot study, we used a nominal cutoff of 17 years and identified 84 at-risk families, in which the PV represented new health information for approximately 80% (66 of 84). Assuming a consistent outcome across the remaining untested Australian population, with upscaling, we estimate that up to 500 more PVs could be found with an expanded program in this country. Given that guidelines for genetic testing in ovarian cancer in most Western countries have been broadened only within the past decade, there remains a window of opportunity to reduce the risk of cancers in at-risk family members. With a high level of engagement of NOK-referral individuals, consideration should be given to obtaining access to subsidized clinical testing of archival cancer tissue for this group. We note that the approach used here may also be applied to other cancer types, including triple-negative breast cancer, which has a high rate of *BRCA1*/*2* germline PVs, and more broadly to solid organ malignancies with a strong association between histology and the presence of inherited susceptibility genes.

**TABLE 2.**
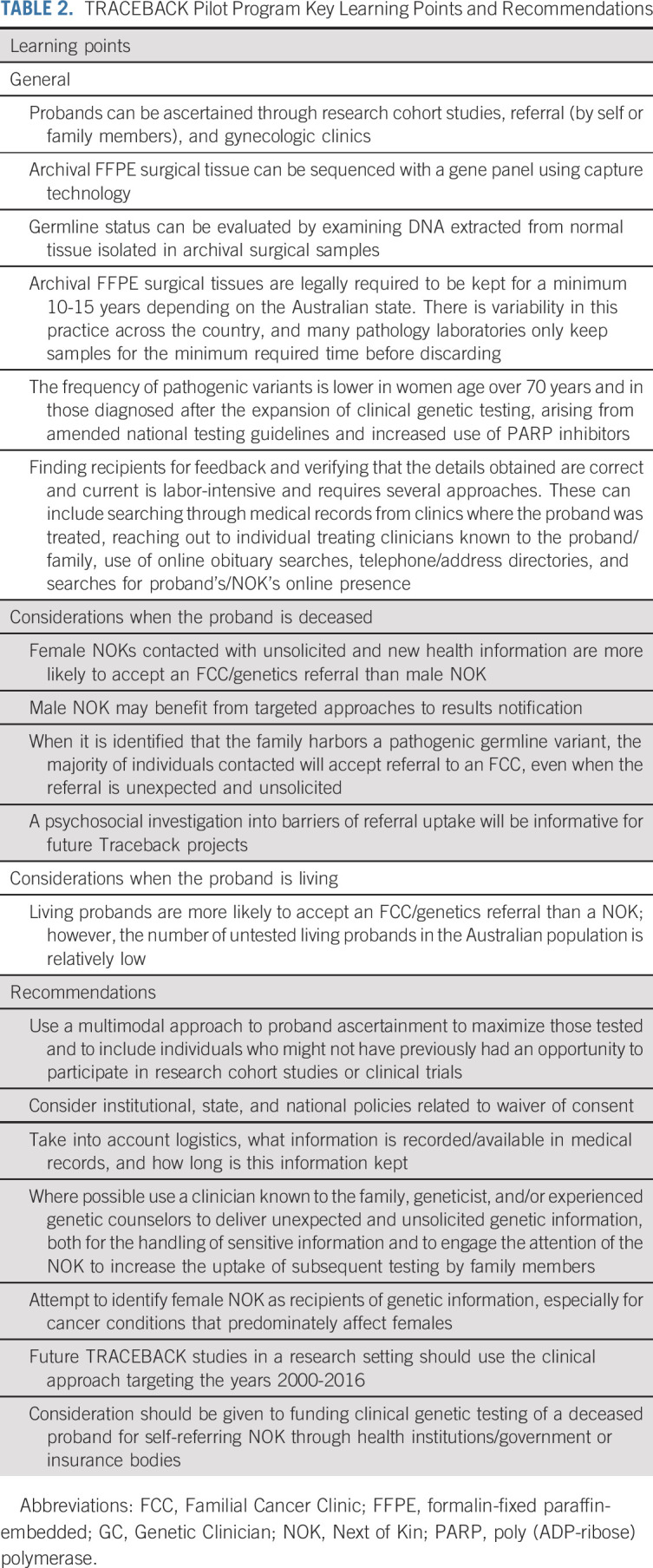
TRACEBACK Pilot Program Key Learning Points and Recommendations
